# Comparative analysis of RNA regulatory elements of amino acid metabolism genes in Actinobacteria

**DOI:** 10.1186/1471-2180-5-54

**Published:** 2005-10-03

**Authors:** Alexander V Seliverstov, Harald Putzer, Mikhail S Gelfand, Vassily A Lyubetsky

**Affiliations:** 1 Institute for Information Transmission Problems, RAS, Bolshoi Karetny pereulok 19, Moscow, 127994, Russia; 2 Institut de Biologie Physico-Chimique, CNRS UPR9073, 13, rue P. et M. Curie, 75005 Paris, France

## Abstract

**Background:**

Formation of alternative structures in mRNA in response to external stimuli, either direct or mediated by proteins or other RNAs, is a major mechanism of regulation of gene expression in bacteria. This mechanism has been studied in detail using experimental and computational approaches in proteobacteria and Firmicutes, but not in other groups of bacteria.

**Results:**

Comparative analysis of amino acid biosynthesis operons in Actinobacteria resulted in identification of conserved regions upstream of several operons. Classical attenuators were predicted upstream of *trp *operons in *Corynebacterium *spp. and *Streptomyces *spp., and *trpS *and *leuS *genes in some *Streptomyces *spp. Candidate leader peptides with terminators were observed upstream of *ilvB *genes in *Corynebacterium *spp., *Mycobacterium *spp. and *Streptomyces *spp. Candidate leader peptides without obvious terminators were found upstream of *cys *operons in *Mycobacterium *spp. and several other species. A conserved pseudoknot (named LEU element) was identified upstream of *leuA *operons in most Actinobacteria. Finally, T-boxes likely involved in the regulation of translation initiation were observed upstream of *ileS *genes from several Actinobacteria.

**Conclusion:**

The metabolism of tryptophan, cysteine and leucine in Actinobacteria seems to be regulated on the RNA level. In some cases the mechanism is classical attenuation, but in many cases some components of attenuators are missing. The most interesting case seems to be the *leuA *operon preceded by the LEU element that may fold into a conserved pseudoknot or an alternative structure. A LEU element has been observed in a transposase gene from *Bifidobacterium longum*, but it is not conserved in genes encoding closely related transposases despite a very high level of protein similarity. One possibility is that the regulatory region of the *leuA *has been co-opted from some element involved in transposition. Analysis of phylogenetic patterns allowed for identification of ML1624 of *M. leprae *and its orthologs as the candidate regulatory proteins that may bind to the LEU element. T-boxes upstream of the *ileS *genes are unusual, as their regulatory mechanism seems to be inhibition of translation initiation via a hairpin sequestering the Shine-Dalgarno box.

## Background

Formation of alternative structures in 5'-leader regions of mRNAs is emerging as a major mechanism of gene regulation. There exist several possible variants of this mechanism whose common feature is the competition between two structures, one of which represses gene expression via premature termination of transcription or inhibition of translation initiation (reviewed in [1-6]). The energetically or kinetically more favourable structure forms by default, whereas the other one is stabilized by binding of a regulatory protein, tRNA, or a small cofactor, or is formed co-transcriptionally, as in classical attenuators.

RNA regulatory elements have been studied mainly in gamma-proteobacteria (*Escherichia coli*) and firmicutes (*Bacillus subtilis*). Computational analysis also has been mainly restricted to proteobacteria [7,8] and firmicutes [9-12]. Recently a new class of regulatory elements, riboswitches, has been described. These elements are highly conserved and were found in all major taxa of bacteria, as well as in some eukaryotes and archaea [13,14]. Comparative genomic analysis has played a major role in the discovery and analysis of T-boxes [9,15] and most riboswitches (reviewed in [4,5]). Several groups performed large-scale search for new RNA regulatory structures [16,17]. Analysis of RNA-based regulation often leads to non-trivial functional assignments for hypothetical genes and filling gaps in metabolic reconstruction (e.g. [11,14,18,19]).

Here we performed comparative analysis of candidate RNA regulatory elements in genomes of Actinobacteria. There are few known attenuators in these genomes. Those that have been experimentally studied are attenuators of the *trp *operons in *Corynebacterium glutamicum *[20] and *Streptomyces venezuelae *[21]. Studies of attenuator-like structures upstream of the *ilvB *and *leuA *genes of *Streptomyces coelicolor *produced somewhat ambivalent results. Indeed, although candidate leader peptides and alternative RNA structures were found upstream of the *ilvB *and *leuA *genes, reminiscent of the classical attenuators, the mutation analysis demonstrated that the regulatory mechanism is not attenuation in the strict sense: mutations in candidate regulatory codons in the leader peptide of the *ilvB *gene had no effect on regulation, and, although mutations in the leader peptide of *leuA *had some effect, it was not consistent with classical attenuation [22]. Computational analysis identified several types of riboswitches: THI-elements [14], RFN-elements [18], B12-elements [19], all of them regulating genes of cofactor metabolism by sequestering the Shine-Dalgarno box and start codon, and interfering with initiation of translation.

## Results and discussion

Following an approach described previously [8], we systematically analysed the upstream regions of amino acid biosynthesis and aminoacyl-tRNA synthetase operons. Candidate regulatory structures were found upstream of genes involved in tryptophan, cysteine, and leucine metabolism. Candidate T-boxes were observed upstream of isoleucyl-tRNA synthetase genes. No conserved structures were observed upstream of genes from other amino acid biosynthesis pathways.

### Tryptophan

**Figure 1:**
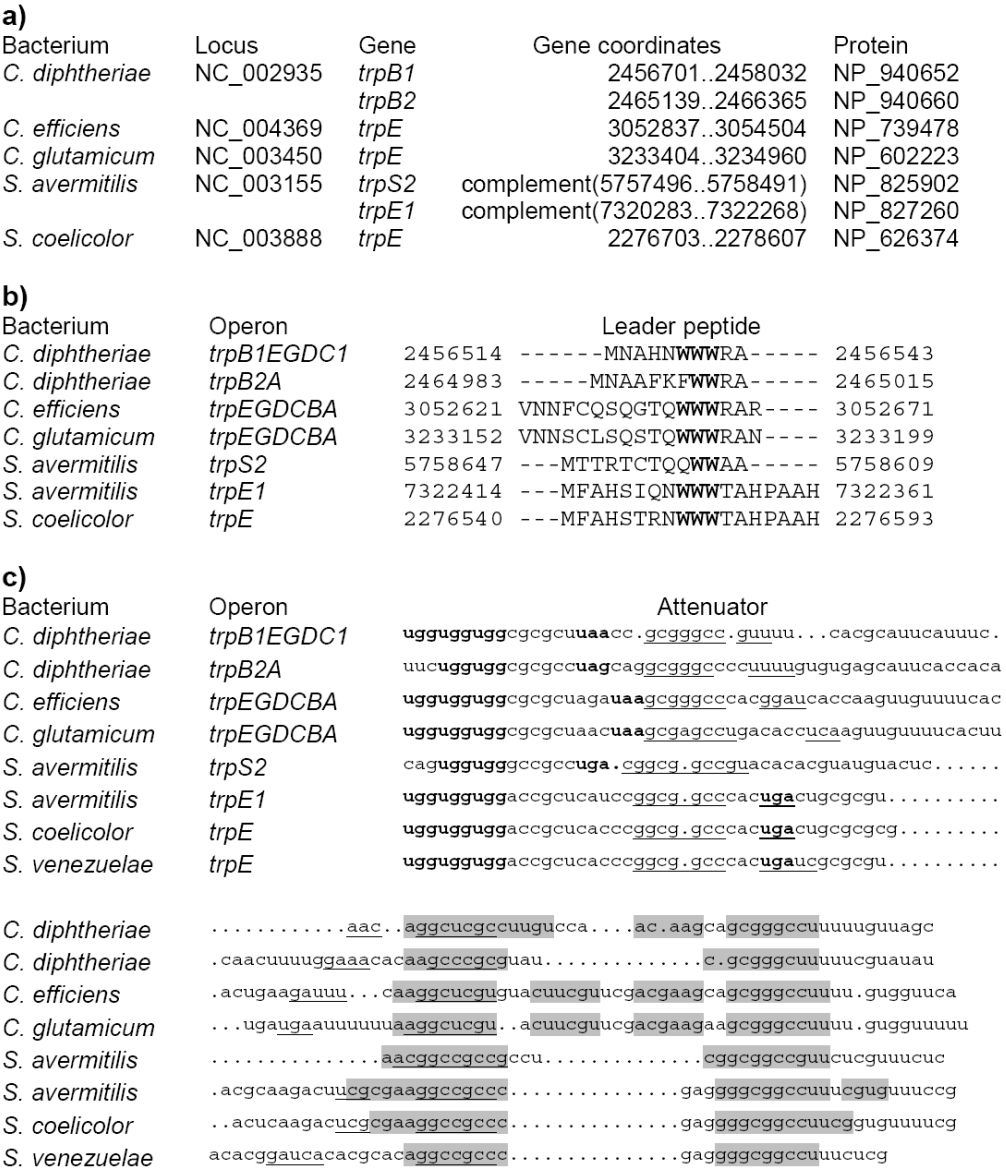
**Leader peptides and candidate attenuators upstream the *trp *operons in *Corynebacterium *and *Streptomyces *spp. **a) Coordinates and protein identifiers of the first genes in the operons. b) Alignment of the leader peptides. The numbers denote genome positions of the aligned fragments. c) Alignment of the attenuators. Tryptophan and stop codons are shown in bold. The terminator hairpins are highlighted in grey, the antiterminator hairpins are underlined. The alignment contains fragments between the tryptohan codons and the terminator hairpin followed by poly-U-tracts. The numbers denote genome positions of the aligned fragments.

The *trp *operons are preceded by classical candidate attenuators in all considered genomes of *Corynebacterium *spp. and *Streptomyces *spp. (Fig. 1). The leader peptides have double or triple repeats of regulatory UGG codons. All terminators are GC-rich and followed by poly-U-tracts. The antiterminator and terminator hairpins in all genomes contain complementary triples gGCC-rGCy-GGCC where absolutely conserved positions are set in capitals. This is analogous to the situation in proteobacteria, where the patterns involved in multiple interactions within attenuators are conserved at large evolutionary distances [8]. In *C. diphteriae*, candidate attenuators were found upstream of both biosynthetic operons *trpB*_1_*EDGC *and *trpB*_2_*A*. A candidate attenuator was found upstream of the tryptophanyl-tRNA synthetase gene *trpS*_2 _in *S. avermitilis*.

### Cysteine

**Figure 2:**
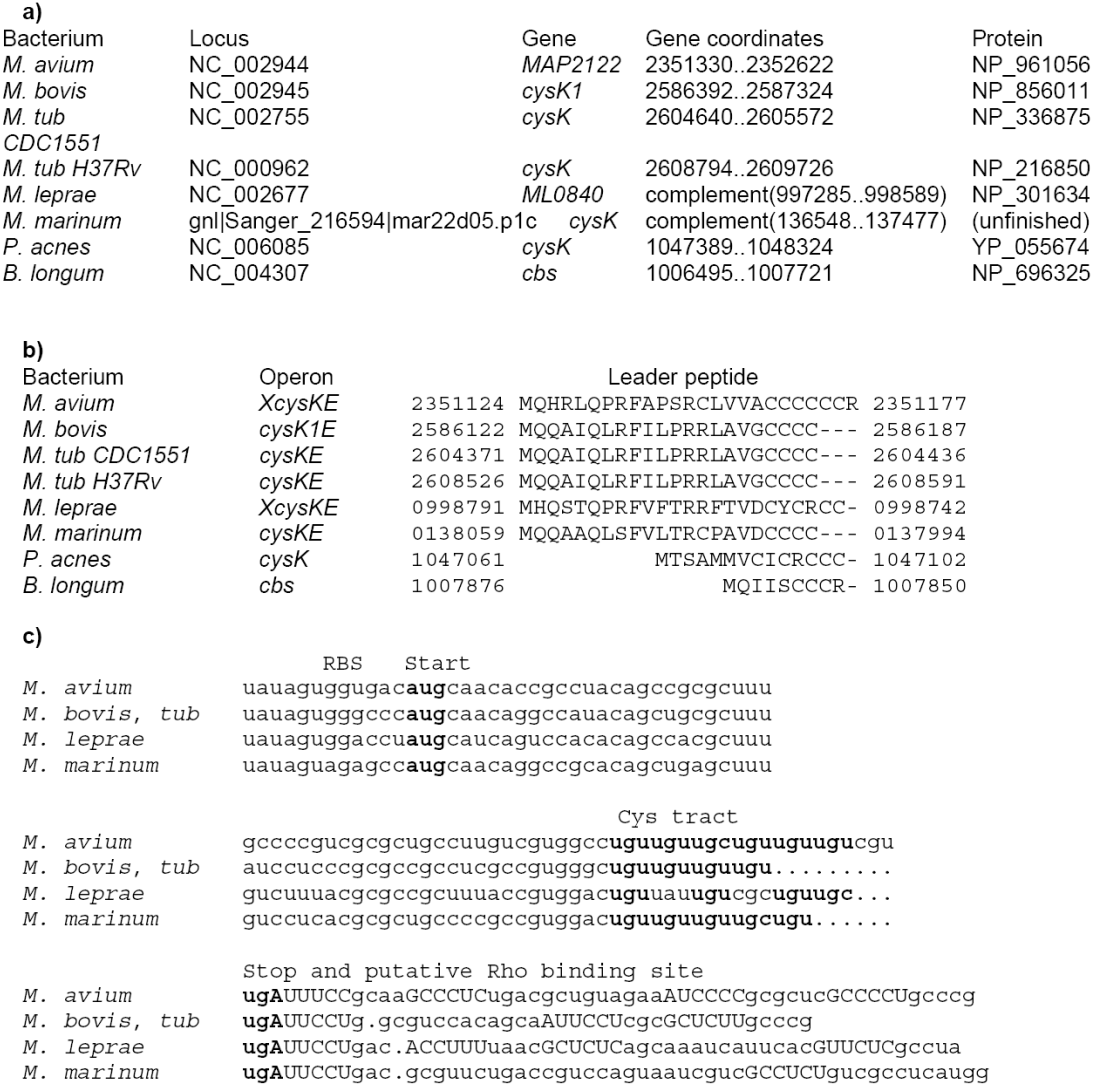
**Leader peptides upstream the *cys *operons in *Mycobacterium *spp. and *P. acnes *and *cbs *operon in *B. longum*. **a) Coordinates and protein identifiers of the first genes in the operons. b) Alignment of the leader peptides. The numbers denote genome positions of the aligned fragments. c) DNA alignment of the leader peptide genes. Start, cysteine and stop codons are shown in bold; candidate Rho-binding sites are shown in capitals.

The upstream regions of the *cys *operon in *Mycobacterium *spp. and *Propionibacterium acnes *and the *cbs *gene of *Bifidobacterium longum *contain short open reading frames encoding candidate leader peptides with runs of cysteine codons near the stop codon (Fig. 2a). The upstream regions of *Mycobacterium *spp. are very similar and can be aligned (Fig. 2b). However, they do not contain any conserved hairpins that could serve as terminators of transcription. One possibility is that this region contains rho-dependent terminators similar to the situation in the tryptophanase operon *tna *of *E. coli *[23]. Indeed, *Mycobacteium *spp. have few rho-independent terminators [24,25]. On the other hand, all *Mycobacterium *genomes contain the components of the rho-dependent termination mechanism, *rho, nusG, nusA, nusB*. The region between the candidate leader peptide ORFs and the first genes in the *cys *operons contain polyY motifs that could serve as Rho-binding sites [26-28]. However, these motifs are not conserved, and thus this prediction is rather weak.

The cysteine operons in *M. avium *and *M. leprae *contain additional hypothetical genes, *MAP2122 *and *ML0840 *respectively, that are 62% identitical but have no other reliable homologs.

### Leucine

**Figure 3:**
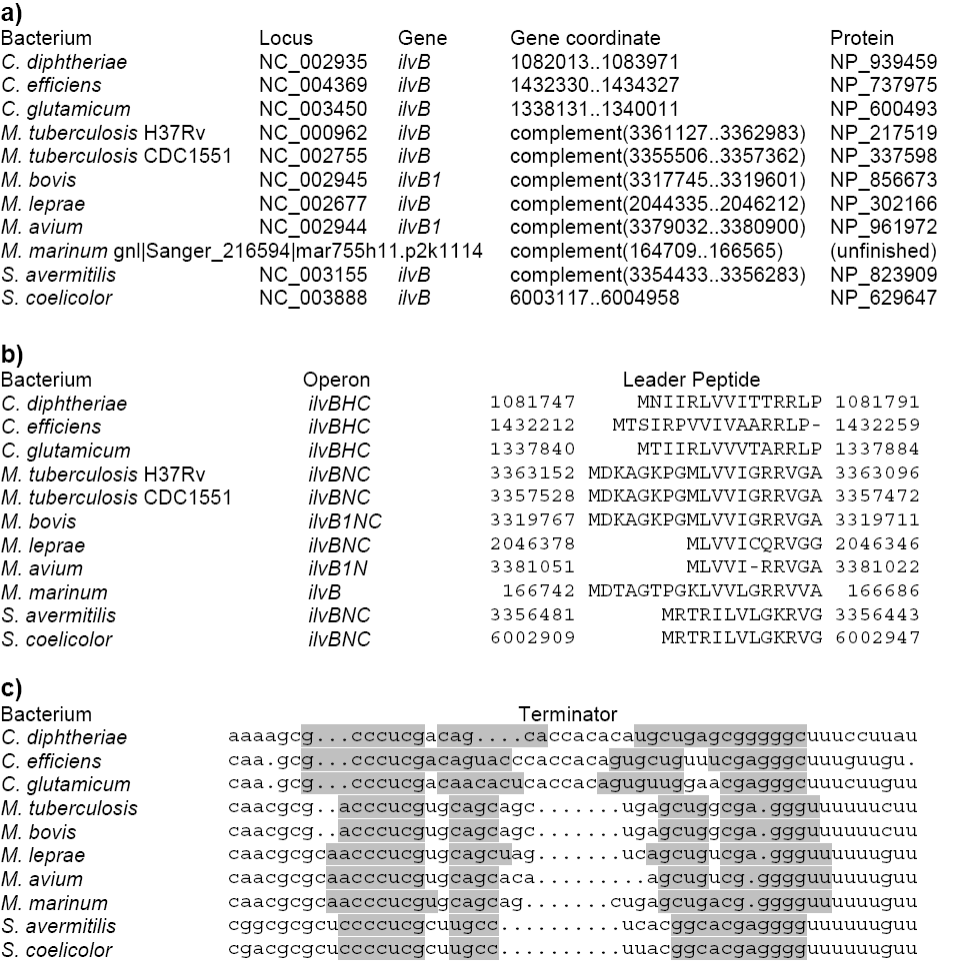
**Candidate leader peptides and terminators upstream the *ilv *opreron in Actinobacteria. **a) Coordinates and protein identifiers of the first genes in the operons. b) Alignment of the leader peptides. The numbers denote genome positions of the aligned fragments. c) Alignment of the terminators. The terminator hairpins are highlighted in grey.

The upstream regions of the *ilvB *genes (operons *ilvBNC, ilvBHC, ilvBserA*_1_) in *Corynebactecterium, Mycobacterium, Streptomyces *species contain short ORFs with runs of isoleucine, valine and leucine codons overlapping the candidate terminator hairpins followed by polyU-runs (Fig. 3). However, the exact mode of regulation is not clear, as experimental substitution of possible regulatory codons upstream of the *ilvBNC *operon in *S. coelicolor *had no effect on regulation or expression of *ilvB *[23].

**Figure 4:**
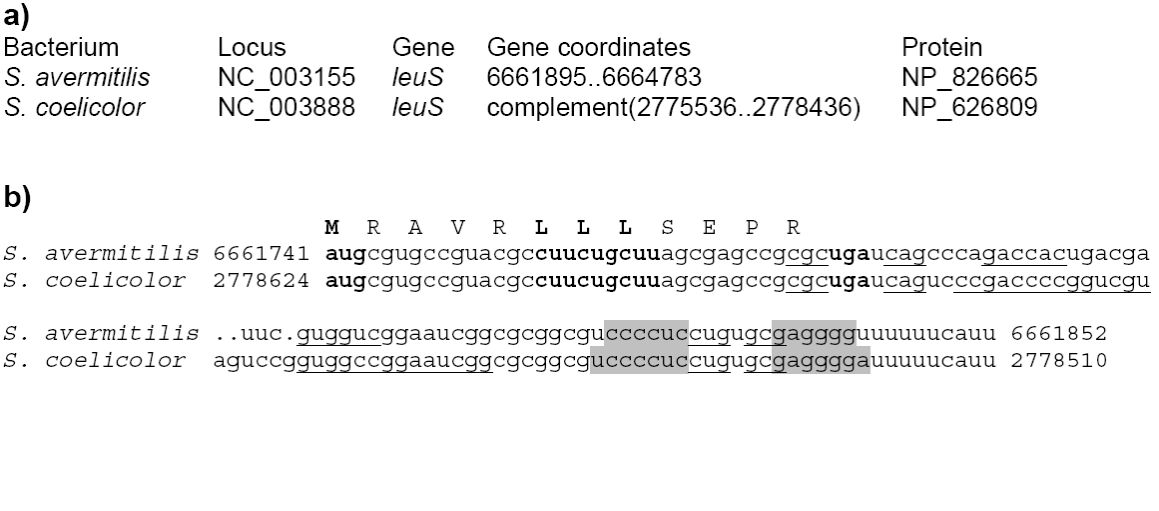
**Candidate attenuators upstream the *leuS *opreron in *Streptomyces *spp. **a) Coordinates and protein identifiers of the *leuS *genes. b) Alignment of the attenuators. Start, leucine and stop codons are shown in bold. The terminator hairpins are highlighted in grey, the antiterminator hairpins are underlined. The alignment contains fragments between the leader peptide ORFs and the terminator hairpin followed by poly-U-tracts.

Classical candidate attenuators were found upstream of *leuS *(leucyl-tRNA-synthetase) in *S. avermitilis *and *S. coelicolor. *Each of them contains an ORFs encoding the leader peptide, as well as the antiterminator and terminator hairpins (Fig. 4).

**Figure 5:**
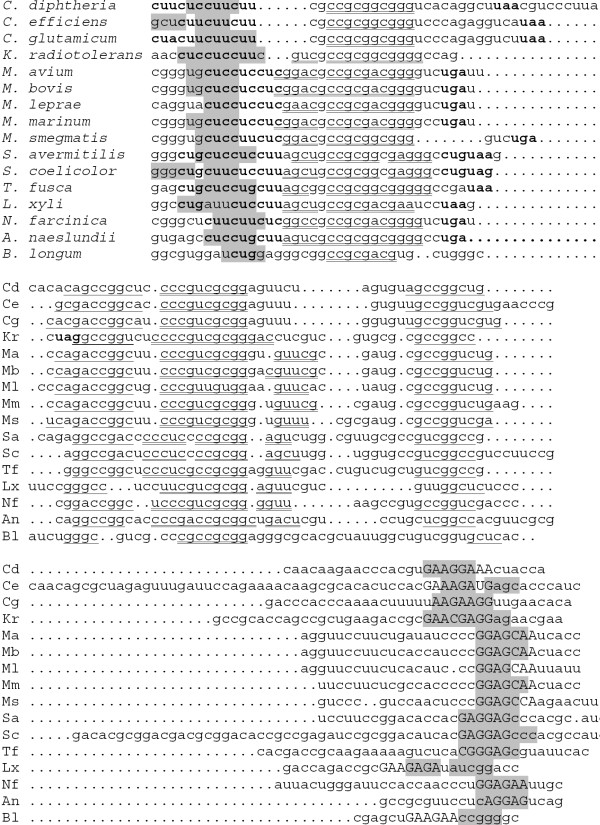
**Alignment and RNA secondary structures of the *leuA *upstream regions (LEU elements). **The stem at the base is highlighted in grey, helices forming the pseudoknot are underlined and double underlined, leucine and stop codons are set in bold, the candidate Shine-Dalgarno boxes of the *leuA *are set in capitals. The last sequence is that of the transposase from *B. longum *(see the text). Sequences for *M. bovis *(Mb) and *M. tuberculosis *spp. (Mt and Rv) coincide.

**Figure 6:**
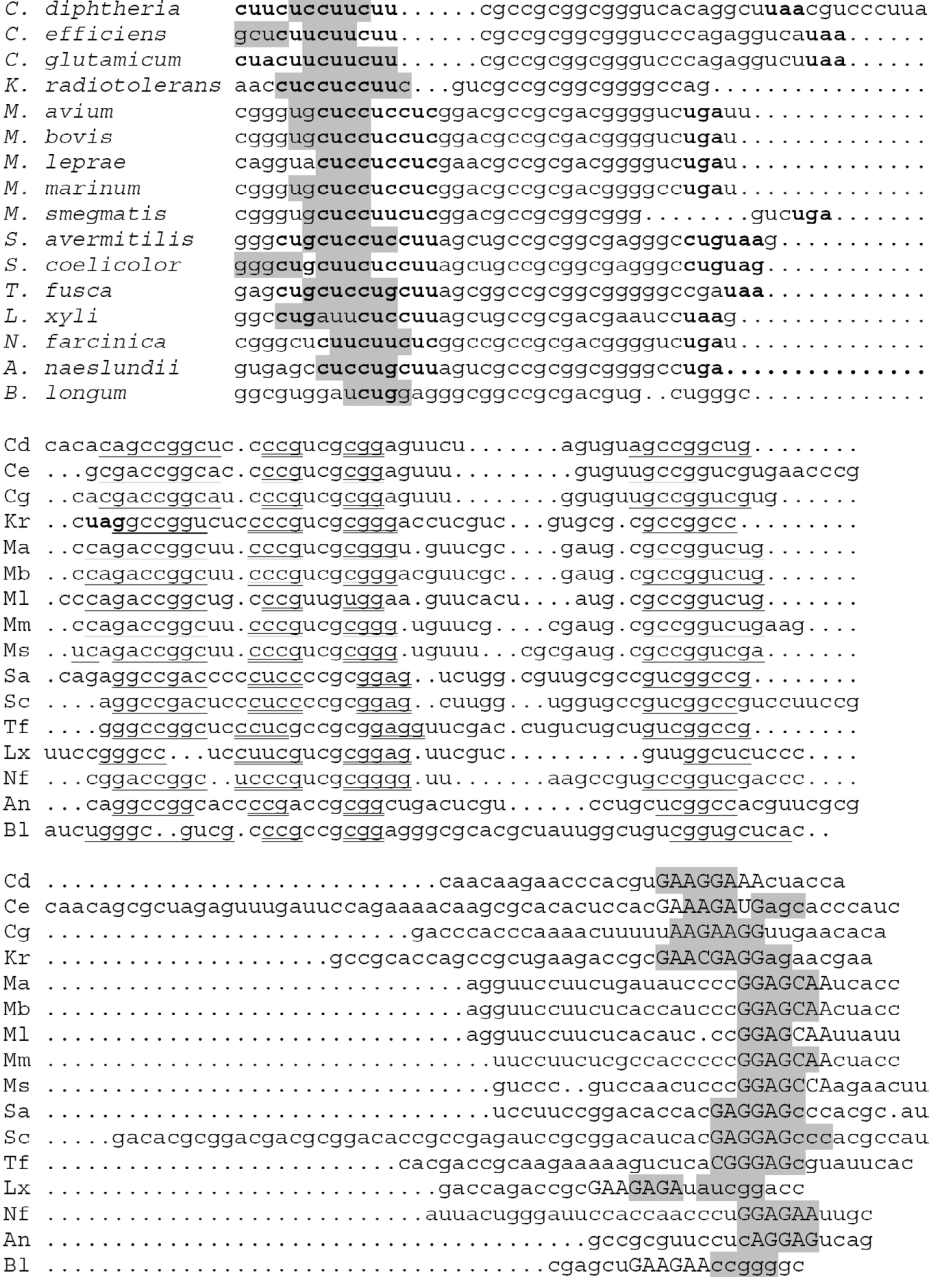
**Alternative RNA secondary structure in LEU elements. **The stem at the base is highlighted in grey, two internal helices are underlined and double underlined, other notation as in Fig 5.

**Figure 7:**
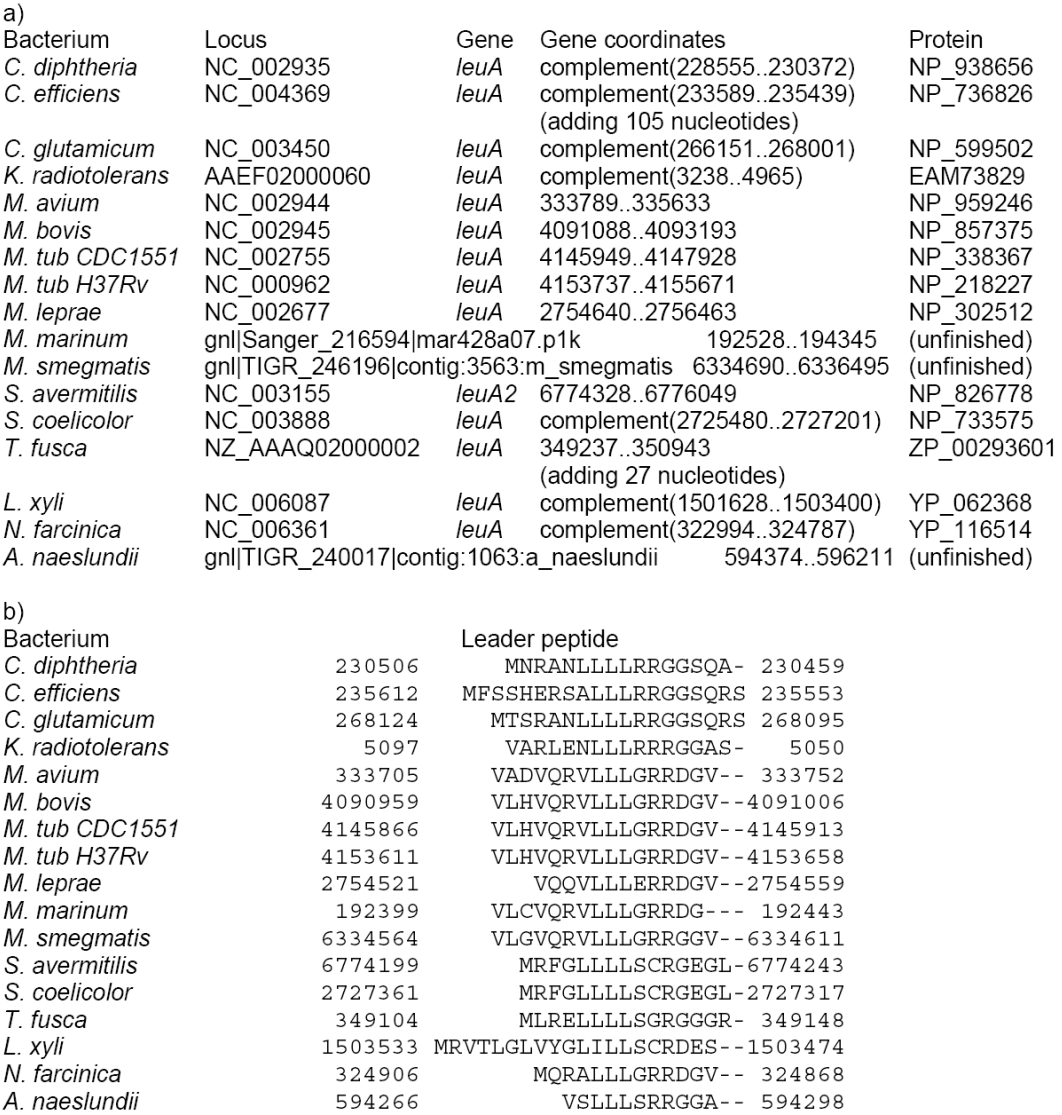
Candidate leader peptides in the LEU elements.

**Figure 8:**
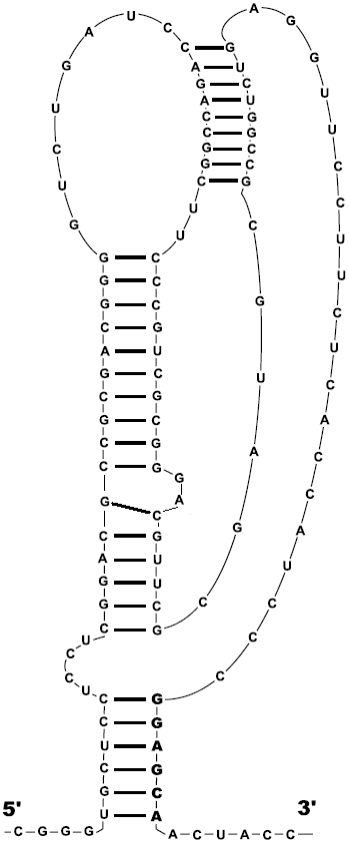
**Candidate RNA pseudoknot upstream of the *leuA *operon in *M. bovis*. **The corresponding alignment is given Fig. 5. Boldface: the candidate Shine-Dalgarno box.

Sequences upstream of the isopropylmalate synthase genes *leuA *contain a number of candidate regulatory sequences, together named the LEU element (Fig. 5, 6). Firstly, there is an upstream ORF encoding a candidate leader peptide with a run of leucine codons (Fig. 7). Secondly, this region may fold into a pseudoknot with an additional stem at its base formed by pairing of the leucine codon run with the Shine-Dalgarno box of the *leuA *gene (Fig. 5, 8). Finally, the same region may form an alternative hairpin with the same base stem (Fig. 6).

**Figure 9:**
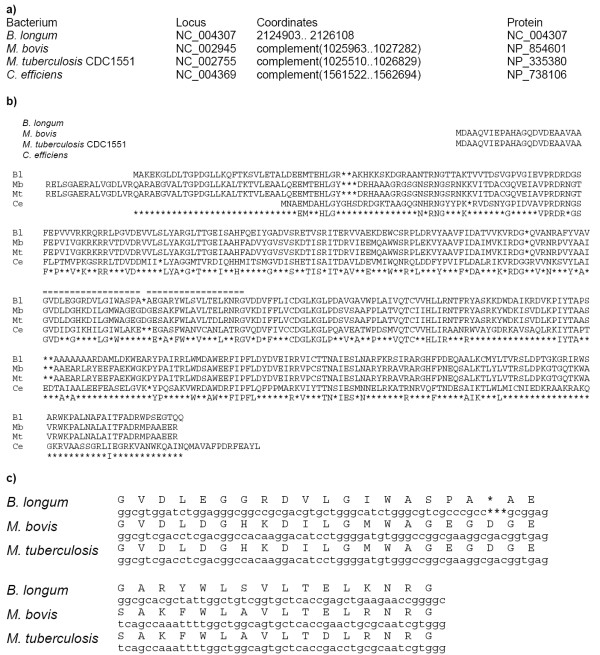
**Multiple alignments of transposases. **a) Coordinates and protein identifiers of putative transposases. b) Protein alignment. The fragment marked by the double line above corresponds to the *B. longum *fragment homologous to candidate pseudoknot and shown in the last line of Fig. 5. c) Nucleotide alignment of the region shown by the double line in (b).

A similar pseudoknot was found in *B. longum *within a gene encoding a transposase. The latter is homologous to the IS1554 transposase of *M. tuberculosis *and *M. bovis *(66% identity), a putative transposase in *C. efficiens *(40% identity), putative IS256 family transposases of *S. avermitilis *(31% identity), hypothetical protein MAP2274 of *M. avium *(29% identity), and some other putative transposases from *B. longum*, *C. efficiens, M. tuberculosis, M. bovis, R. xylanophilus, S. avermitilis, S. coelicolor *(Fig. 9a). However, only the *B. longum *transposase contains a fragment that may fold into the pseudoknot (Fig. 9b), whereas other transposases, although highly similar on the protein level in the corresponding region, contain a number of non-complementary mismatches in synonymous codon positions and thus have lost the pseudoknot folding potential.

### T-boxes

**Figure 10:**
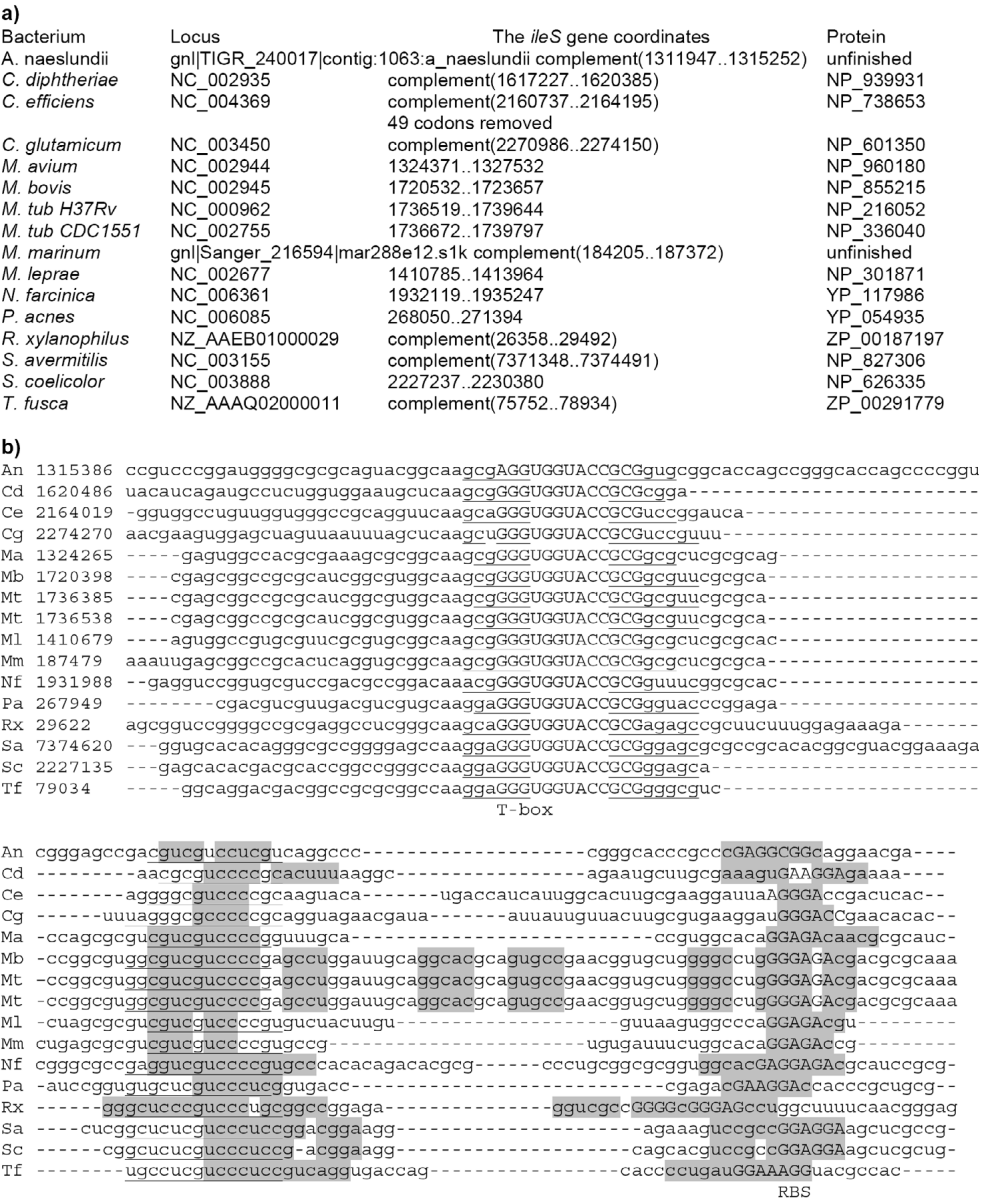
**Multiple alignment of T-box structures upstream of the *ileS *genes. **a) Coordinates and protein identifiers of the *ileS *genes. b) Nucleotide alignment of the 5' untranslated regions. T-box hairpins are underlined and T-box sequences are set in capitals. The sequestor hairpin is shaded in grey. Candidate Shine-Dalgarno boxes are set on capitals. Anti-sequestor hairpins are set in bold.

Candidate T-box structures were found upstream of the *ileS *genes from several Actinobacteria. They are unusual, as instead of terminators, they contain hairpins sequestering the Shine-Dalgarno boxes of the *ileS *genes (Fig. 10). Thus it is likely that the regulatory mechanism involves inhibition of translation initiation. To our knowledge, this is the first example of a T-box acting on the level of translation.

## Conclusion

Candidate regulatory elements were found upstream of genes involved in the tryptophan, cysteine and branched chain amino acids metabolism. No conserved RNA regulatory structures were observed upstream of histidine, threonine, phenylalanine, tyrosine, arginine, lysine, methionine operons, although orthologous genes involved in the latter pathways are regulated on the RNA level in other species: methionine and lysine by the S-box and L-box riboswitches respectively [3-5], histidine, threonine and phenylalanine by attenuators [7,8], tyrosine and arginine by T-boxes [12].

Attenuators of the classical type were observed upstream of the aminoacyl-tRNA-synthetase genes *trpS *and *leuS *in some *Streptomyces *genomes, similar to those observed in gamma-proteobacteria, (e.g. the *pheST *operon) [7]. In contrast, in Firmicutes, most aminoacyl-tRNA-synthetase genes are regulated by tRNA-dependent antitermination (T-boxes) and none by classical attenuation [2,9,15]. No classical T-boxes were found in Actinobacteria, but unusual T-boxes, possibly regulating initiation of translation, were observed upstream of the *ileS *genes in several genomes.

Despite the presense of conserved leader peptides upstream of some cysteine and leucine operons, the mode of regulation is unknown, as other attenuator elements are missing. One possible explanation is that attenuation of the *cys *operons in *Mycobacterium *spp. and *P. acnes *and the *cbs *operon in *B. longum *involves Rho-dependent termination, similar to the *tna *operon of *E. coli *[23,29].

The most interesting case seems to be that of the *leuA *genes. The upstream regions of these genes contain several conserved elements (referred to as the LEU element) that can be interpreted in different ways. There are some architectural similarities with riboswitches, in particular, a compact structure with a stem at the base [5,30,31]. The latter is formed by interaction of a run of leucine codons and the Shine-Dalgarno box. Indeed, Actinobacteria seem to be the only taxonomic group where the base stems of riboswitches directly overlap the translation initiation site, without additional regulatory hairpins [5]. However, the LEU element differs from all known riboswitches, as the alignment of LEU elements does not contain conserved unpaired nucleotides that would be involved in tertiary interactions and form the ligand-binding pocket, as in the purine riboswitches whose spatial structure has been resolved [30,31] and in other riboswitches [5]. Thus direct binding of a small molecule to LEU elements seems unlikely. On the other hand, there is experimental evidence that mutations in the leucine codons do not influence the regulation [22] and thus classical attenuation involving translation of a leader peptide also is an unlikely mechanism of regulation.

The above considerations make it likely that the LEU element is a binding site of some regulatory protein. To test for this possibility, we compared the pattern of phylogenetic distribution of LEU elements to phylogenetic distributions of all actinobacterial genes. The closest phylogenetic pattern was observed for orthologs of ML1624 from *M. leprae*: homologs of this protein with E-values <10^-170 ^were found in all genomes containing LEU elements, but not outside Actinobacteria. The only unexplained fact is the presence of a homolog with the E-value ~10^-108 ^in *P. acnes*, which does not have a LEU element. The structure of the ML1642 protein is consistent with an RNA-binding regulatory role, as the protein contains an N-terminal DEAD-box helicase domain (ProFam family PF00270, E-value 3.6·10^-6^) that may be involved in unwinding of nucleic acids.

An additional enigma is the presence of a LEU element-like sequence within a transposase gene. On the other hand, it may be a clue to the origin of LEU elements. One possibility is that the *B. longum *transposase represents an ancestral state where the LEU element was involved in maintenance or regulation of transposition. Situations when a regulatory site occurs within a regulatory and/or regulated gene are not very common, but they happen in mobile elements [32]. Other transposase genes may have lost the ability to form this structure due to mutations; notably, the protein sequence has not changed much (Fig. 9), as most mutations occurred in synonymous codon positions. A plausible scenario is that the transposase gene was inserted upstream of the *leuA *gene in the ancestral actinobacterial genome. The main fraction of the coding sequence was subsequently deleted, whereas the structural element was co-opted for regulation of the downstream *leuA *gene.

## Methods

Genomes of Actinobacteria *Actinomyces naeslundii *(An), *Bifidobacterium longum *(Bl), *Corynebacterium diphtheriae *(Cd), *Corynebacterium efficiens *(Ce), *Corynebacterium glutamicum *(Cg), *Kineococcus radiotolerans *(Kr), *Leifsonia xyli *(Lx), *Mycobacterium avium *(Ma), *Mycobacterium bovis *(Mb), *Mycobacterium leprae *(Ml), *Mycobacterium marinum *(Mm), *Mycobacterium smegmatis *(Ms), *Mycobacterium tuberculosis *(Rv and Mt), *Nocardia farcinica *(Nf), *Propionibacterium acnes *(Pa), *Rubrobacter xylanophilus *(Rx), *Streptomyces avermitilis *(Sa), *Streptomyces coelicolor *(Sc), *Thermobifida fusca *(Tf), *Tropheryma whipplei *(Tw) were downloaded from the NCBI web site. We also used sequences of *Streptomyces venezuelae *(Sv) from [21].

Candidate operons were defined as chains of genes transcribed in the same direction with intergenic regions not exceeding 150 nucleotides. Multiple alignments of genes were used to verify and, if necessary, revise annotated gene starts [33]. The revisions included adding 105 nucleotides (35 codons) to the *leuA *gene from *C. efficiens*, adding 27 nucleotides (9 codons) of the *leuA *gene from *T. fusca*, and removing 147 nucleotides (49 codons) of the *ileS *gene from *C. efficiens*.

RNA sequence and structure alignments were constructed using MultAlign (A.A. Mironov, personal communication) and the program GL [34]. Search for RNA structural patterns was performed using the PAT program (A.V.Seliverstov, unpublished). Search for conserved sequence fragments was done using the CLIQUE program [35]. Multiple protein sequence alignments were constructed using MultAlign.

## Authors' contributions

AVS and VAL developed algorithms. AVS wrote the programs and performed sequence analysis. HP and AVS identified translational T-boxes. AVS, VAL, and MSG analyzed LEU elements. AVS and MSG performed functional annotation and wrote the paper. VAL and MSG conceived and supervised the project.
